# Doctors with mental health difficulties and ADHD

**DOI:** 10.1093/occmed/kqae139

**Published:** 2025-01-23

**Authors:** B Perera, Z Al-Najjar

**Affiliations:** University College London, 149 Tottenham Ct Rd, London W1T 7NF, UK; NHS Practitioner Health, 18 Wandsworth Rd, London SW8 2JB, UK

## Abstract

**Background:**

There is growing interest in understanding neurodevelopmental disorders such as Attention-deficit/hyperactivity disorder (ADHD) among doctors. However, the current understanding of ADHD and its association with mental well-being in doctors is limited.

**Aims:**

This study investigated the significance of ADHD among doctors with mental health difficulties accessing a national mental health service for doctors in England.

**Methods:**

Electronic records from 2877 doctors seeking mental health care through the National Health Service Practitioner Health service were analysed. Demographic data, psychopathology scales (PHQ-9 for depression, GAD-7 for anxiety, Core-10 for psychological well-being) and ADHD screening using ADHD Self-Report Scale (ASRS) were examined. Analyses were conducted to explore associations between ADHD screening, demographic variables and co-existing mental health disorders.

**Results:**

The study revealed that over one-third (35%) of doctors in this study sample screened positive for ADHD using the ASRS questionnaire. The male-to-female ratio for screened positive was 1.1:1. The number of doctors screening positive for ADHD reduced with age. A substantial portion of doctors who screened positive for ADHD also exhibited symptoms of co-existing mental health disorders such as anxiety and depression.

**Conclusions:**

This study highlights that assessments for ADHD among doctors presenting with mental health difficulties can be important and relevant. Validated screening tools can be used in this process. The high rate of psychopathology among those who screened positive for ADHD in this study sample indicates the need for detailed assessments to understand the complex dynamic of ADHD symptoms and psychiatric disorders. Recognizing ADHD is important as treatments are different to other psychiatric disorders.

## Teaser

Understanding factors associated with mental health difficulties among doctors is important for improving care and to reduce their distress. While studies show a strong association between ADHD and mental health disorders, this has not been explored in doctors presenting with mental health problems. This study found over one-third of doctors with mental health problems screened positive for ADHD, highlighting the need to investigate neurodevelopmental disorders such as ADHD in this group.

**Key learning points**

What is already known about this subject:

- ADHD is a common neurodevelopmental disorder.
- It is associated with a high burden of mental disorders.
- The association between ADHD and mental well-being in doctors is not known.

What this study adds:

- One-third of doctors in this study seeking mental health care screened positive for ADHD
- Doctors who screened positive for ADHD also tend to score high for anxiety and mood symptoms, and low for psychological well-being.
- The percentage of doctors screening positive for ADHD reduced with increasing age.

What impact this may have on practice or policy:

- There is a need to develop ADHD diagnostic and treatment pathways for doctors presenting with mental health problems.

## INTRODUCTION

Mental ill health has been cited as the most common reason for absence due to sickness in the National Health Service (NHS) in the UK [1]. It is estimated that an NHS healthcare employee is two to three times more likely to require sickness leave for mental ill health compared to an average UK employee [1]. Among doctors, the prevalence rate is 17–52% for psychiatric morbidities [2]. Individual personality traits, psychological impact of work and the recent global pandemic have been suggested as some of the reasons [3,4]. This is a growing concern given that the increased rate of mental disorders is associated with burnout, absence from work, poor work performance and work errors in addition to the significant impacts they can have on a person’s life [5,6].

In the UK, Practitioner Health (PH) is funded by the NHS to provide a confidential mental health service to healthcare professionals. It is a primary care service that provides assessment and treatment for mental health and addiction issues affecting health and social care staff in England and Scotland. As a confidential service, PH attracts a cohort who may otherwise choose not to present to their local mental health services out of concerns about familiarity with staff and confidentiality [7]. PH is staffed by general practitioners, psychiatrists and specialist allied mental health professionals. Referrals to PH are screened by a health professional before assigning to diagnostic and treatment pathways. The pathways include common mental and emotional disorders, anxiety, depression, burnout, complex conditions such as obsessive–compulsive disorder, and bipolar affective disorder. However, currently there is no formalized pathway in PH for the diagnosis and treatment of attention-deficit/hyperactivity disorder (ADHD).

ADHD is a neurodevelopmental disorder that has its origins in childhood causing significant functional impairment due to poor concentration and/or hyperactivity, and impulsivity. The prevalence rate of ADHD in adults in the general population is approximately 2–3% [8]. There has been some emphasis on identifying ADHD in certain professional groups, but the prevalence rate among doctors has not been established [9]. In a survey of 145 US medical schools, ADHD was the most common self-disclosed disability [10]. A cross-sectional study of 487 medical students in Saudi Arabia reported higher rates of ADHD compared to the general population. A similar study among medical students in China demonstrated relatively high rates of ADHD [10,11]. The evidence suggests that the rate of ADHD may be higher among doctors compared to the general population. Therefore, it could be argued that it is important to understand ADHD among doctors. Epidemiological studies have shown the association between ADHD and psychiatric disorders, difficulties in the workplace and burnout, which are important factors to consider in the workforce. Prevalence estimates of depression (42%) and anxiety (44%) are approximately nine times higher among people with ADHD than those without [12]. Understanding the presence of ADHD and its association with mental health difficulties among doctors is important to develop care pathways and to improve outcomes. This study aimed to investigate the significance of ADHD among doctors with mental health difficulties accessing mental health services and to explore associated mental health difficulties.

## METHODS

This naturalistic study used anonymised electronic records gathered by PH. The authors assert that all procedures contributing to this work comply with the ethical standards of the relevant national and institutional committees on human experimentation and with the Helsinki Declaration of 1975, as revised in 2008. Data were managed under NHS Research Ethics Committee approvals (ref. 24/SW/0019). The database contains anonymized routine clinical data from PH in England. Extracted data maintained patient anonymity and all identifiable data were removed. The study covers the period from September 2022 to March 2023.

Data on doctors accessing PH services for mental health difficulties and who consented to use their records for research purposes were used in this study. The data included demographic variables (age, gender, sexual orientation, religion and ethnicity) and psychopathology scales. These include the PHQ-9, GAD-7 and Core-10 questionnaires. The Patient Health Questionnaire (PHQ-9) is a self-administered depression module that reliably measures severity of depression [13]. The 7-item Generalized Anxiety Disorder Scale (GAD-7) is a practical self-report anxiety questionnaire that reliably measures anxiety in the general population [14]. Core-10 is a self-reported measure of psychological distress that reliably measures psychological well-being [15]. A score of 15 and above for the PHQ-9, 15 and above for the GAD-7 and 25 and above for the Core-10 were taken as a positive score. These measures were used in this study as they were routinely collected by PH.

Screening for ADHD was done using the 6-item Adult ADHD Self-Report Scale v1.1 (ASRS) [7]. It consists of six questions assessing hyperactive/impulse and inattention symptoms. For all questions, a grading system of ‘never’, ‘rarely’, ‘sometimes’, ‘often’ and ‘very often’ is used to describe the frequency of occurrence. In the first three items, a score of one point was assigned for a frequency of at least ‘sometimes’ or more. The remaining three items carry a score of one point for answers reporting a frequency of at least ‘often’ or more. Participants with a total score of four or greater were considered to screen positive for ADHD [16]. ASRS is a reliable and valid screening tool with a sensitivity of 68.7% and specificity 99.5%. Its positive predictive and negative values are 0.94 and 0.24, respectively [16]. It is time-efficient and designed to be used for unsupervized self-reporting of symptoms.

## RESULTS

Of a total of 2941 participants (doctors) in the study sample, some were excluded due to inaccurate date-of-birth recording giving a total of 2877 participants in the final analysis. The majority of the cohort were aged between 30 and 39 years (41%), followed by 40–49 (25%), 20–29 (19%) and 50–59 (13%) with few participants aged 60 years or over (1.57%). More women (73%) than men (27%) used the service. The ethnic composition of the participants was White (65%), Asian (24%), Black (3%) and mixed (4%).

Of the group, 993 (35%) screened positive for ADHD with a score of 4 or above using the ASRS questionnaire (Table 1). Stratifying by age group, a mostly linear trend existed where participants appeared progressively less likely in each age bracket to screen positive for ADHD than their younger counterparts with 37% screening positively in the 20–29 age group versus 0% in the 80–89 age group. An exception was observed in the 70–79 age group, where an upswing of 50% of participants screened positive; however, the sample size was small (*n* = 4).

**Table 1.** ADHD detection rates within sample, stratified by age range

ASRS screening result (*n*)	Age range						
	20–29	30–39	40–49	50–59	60–69	70–79	80–89
Positive	207 (37%)	418 (35%)	247 (34%)	111 (30%)	8 (21%)	2 (50%)	0 (0%)
Negative	346 (63%)	761 (65%)	482 (66%)	260 (70%)	31 (79%)	2 (50%)	2 (100%)

Male participants exhibited a higher likelihood of screening positive for ADHD on the ASRS, with 38% compared to 33% of female participants. The prevalence rate of screening positive for ADHD was consistent across participants from White, Asian, Black and mixed ethnic groups (range: 32–35%) (Table 2). There was a higher prevalence of screening positive for ADHD in participants of Latino/Hispanic ethnicity (*n* = 3, 60%) and the ethnic category of ‘other’ (*n* = 47, 51%); however, the sample size for Latino/Hispanic ethnicity group was small.

**Table 2.** ADHD detection rates within sample, stratified by ethnicity

Ethnicity	ASRS screen positive (*n*)
Asian	226 (33%)
Black	31 (33%)
Latino/Hispanic	3 (60%)
Mixed	38 (36%)
Other	47 (52%)
White	638 (34%)
Not disclosed	10 (31%)

Analysis of the symptoms of ADHD among those who screened positive for ADHD revealed that 97% recorded ‘trouble wrapping up the final details of a project once the challenging parts have been done’ compared to 64% of the total participants. 95% of those who screened positive also reported ‘difficulties getting things in order when a task requires organization’. Only 65% of the total participants screened positive for this. The third highest scored symptom in ASRS was ‘avoiding or delaying tasks that require a lot of thought’. 94% of those who screened positive for ADHD reported this symptom compared to 52% of the total participants. Only 40% of those who screened positive for ADHD on ASRS recorded ‘feeling overactive’ compared to 27% of the total sample (Table 3).

**Table 3.** ASRS questionnaire results

ASRS statements	Frequency of symptom (*n* = total number of respondents)							
		Not selected	Never	Rarely	Sometimes	Often	Very often	Screened positive
Trouble wrapping up the final details	Total sample	124 (4%)	408 (14%)	515 (18%)	758 (26%)	656 (23%)	416 (14%)	63%
	Screened positive for ADHD	0 (0%)	1 (<1%)	22 (2%)	257 (26%)	341 (34%)	372 (37%)	97%
Difficulty getting things in order when a task requires organization	Total sample	130 (5%)	466 (16%)	424 (15%)	896 (31%)	961 (33%)	0 (0%)	64%
	Screened positive for ADHD	1 (<1%)	9 (1%)	44 (4%)	313 (31%)	323 (33%)	303 (31%)	95%
Problems remembering appointments or obligations	Total sample	129 (4%)	568 (20%)	359 (13%)	951 (33%)	600 (21%)	270 (9%)	63%
	Screened positive for ADHD	2 (<1%)	18 (2%)	114 (11%)	325 (32%)	289 (29%)	245 (25%)	86%
Avoid or delay getting started for tasks that require a lot of thought	Total sample	134 (5%)	149 (5%)	666 (23%)	423 (15%)	801 (28%)	704 (24%)	52%
	Screened positive for ADHD	0 (0%)	1 (<1%)	11 (1%)	53 (5%)	376 (38%)	552 (56%)	94%
Fidget or squirm with your hands or feet when have to sit for long	Total sample	128 (4%)	724 (25%)	425 (15%)	655 (23%)	509 (18%)	436 (15%)	33%
	Screened positive for ADHD	0 (0%)	46 (4%)	123 (13%)	176 (18%)	295 (30%)	353 (35%)	65%
Feeling overly active/like driven by a motor	Total sample	128 (5%)	992 (34%)	287 (10%)	703 (24%)	532 (19%)	235 (8%)	27%
	Screened positive for ADHD	1 (<1%)	109 (11%)	231 (23%)	252 (25%)	205 (20%)	195 (20%)	40%

Among those who screened positive for ADHD, 46% scored high for depression (PHQ-9), 46% scored high for psychological distress (Core-10) and 44% scored high for anxiety (GAD-7) (Table 4).

**Table 4.** ADHD detection rates within sample, stratified by PHQ-9 score, Core-10, GAD-7

	**PHQ-9 score n (%)**		
		≥15	≤14
ASRS screening result	Positive	588 (46%)	405 (25%)
	Negative	683 (54%)	1201 (75%)
	**Core-10 score n (%)**		
		≥25	≤24
ASRS screening result	Positive	256 (46%)	737 (32%)
	Negative	297 (54%)	1587 (68%)
	**GAD-7 score n (%)**		
		≥15	≤14
ASRS screening result	Positive	503 (44%)	490 (28%)
	Negative	641 (56%)	1243 (72%)

## DISCUSSION

As the understanding of mental health difficulties among doctors continues to grow, it is important to identify the various factors that may predispose individuals to mental health problems. The role of ADHD as a neurodevelopmental disorder and its association with mental health problems among doctors has not been studied before. In this study, over a third of doctors presenting to PH with mental health difficulties screened positive for ADHD that is significantly higher than the range of 1–5% reported among medical students in the USA and in the general population [10,17,18].

The gap in the male-to-female ratio for screening positive for ADHD was smaller (1.13:1) in the sample, in contrast to the 2-3:1 ratio reported in the general population [19]. Doctors noticing and becoming more aware of the impact of symptoms of ADHD irrespective of their gender may be a reason for this reduced gap. There was an almost linear, inversely proportional relationship between increasing age and screening positive for ADHD. This may correlate with studies that have shown a syndromic remission (loss of full diagnostic status) of ADHD with increasing age [20,21]. Possible reasons for this include a natural trajectory of ADHD or a reduction in symptoms due to adaptive strategies that may have evolved with increasing age to manage the challenges associated with ADHD. It can also be argued that increasing demands during the early stages of medical training, such as postgraduate exams, may surface ADHD symptoms compared to later in life when such demands are less intense.

While screening positive does not equate to a diagnosis of ADHD, it is worth exploring the high positive rate in this study population. Several factors may contribute to this variability, including overlapping symptoms of ADHD with mental illnesses, demographic differences in the sample population and barriers and delays in obtaining a diagnosis of Adult ADHD.

The higher rate of ‘screen positive for ADHD’ compared to that of studies on medical students may be associated with the presence of psychiatric comorbidity in this sampled population, who had all sought treatment from PH for an underlying mental health problem. There are two possible explanations for this association. The literature indicates that people with ADHD often experience other psychiatric disorders. Therefore, one possible explanation is that the prevalence rate of ADHD among doctors who present with mental health problems to PH is higher than that of doctors without mental health problems [22,23]. If this possibility is correct, it could therefore be postulated that higher scorings in psychopathology rating scales should suggest the need to screen for ADHD when a doctor present with mental health difficulties. This is reflected within our results, where a positive scoring for GAD-7, Core-10 or PHQ-9 was correlated with positive scores for ASRS in over 40% of cases. The predictive value of a diagnosis of anxiety and/or depression for ADHD has been well-documented [24]. Furthermore, understanding the co-existence of ADHD in a person with anxiety or mood disorder is important as it can change the presentation of underlying difficulties, prognosis and response to treatment [25].

Another explanation is that the presence of anxiety or depressive symptoms can lead to symptoms similar to those of ADHD, hence the high rate of screening positive for ADHD with ASRS. For example, a person with high anxiety or depressive symptoms may struggle to concentrate and have problems ‘remembering appointments’ or ‘difficulty getting things in order when a task requires organization’ which are manifestations of ADHD. Similarly, a person may ‘fidget or squirm with their hands or feet’ if they are highly anxious. Due to this complexity, there is a risk of diagnostic overshadowing when clinicians attribute such symptoms to a mental illness or ADHD rather than considering the possibility of co-existence or that one diagnosis presenting as the other. This highlights the complex nature of psychiatric assessment in this patient group, where underlying neurodevelopmental disorders need to be carefully considered when assessing for the presence of mental illnesses.

As with all forms of mental health diagnoses in healthcare professionals, the diagnosis of ADHD may be limited by the presence of various barriers to healthcare such as self and perceived stigma, accessibility issues, negative career implications and confidentiality concerns [26]. People with ADHD may present with non-ADHD symptoms such as with co-existing mental disorders. Therefore, the use of scoring systems such as PHQ-9 and GAD-7 may help to indicate the need for assessment of ADHD among those presenting with mental health problems given the high rate of ‘screened positive for ADHD’ in this population.

To the best of our awareness, this is the first study to screen for ADHD in a large sample of healthcare professionals presenting with mental health difficulties. Understanding ADHD and its presentation is important in specific populations such as this as it may help to address issues related to detection and improve accurate diagnosis and treatment. There are unique challenges faced by qualified healthcare practitioners in clinical practice that may unmask underlying ADHD often leading to significant functional impairments and worsening of mental well-being.

There are several limitations to note. It is unclear whether participants had previously, or at the time of the study, been assessed and treated for ADHD. This could have biased the prevalence rate of ADHD limiting its use as a proxy measure for healthcare professionals. The ASRS, whilst well established, does not have 100% sensitivity and specificity for ADHD and therefore it is possible that ADHD is being under or even over-diagnosed within this population. The detection sensitivity for ADHD was not assessed in this study. A longitudinal prospective study aggregating the results of onward psychiatric referral could account for this. In addition, generalizability of this data globally is limited in that the study specifically assesses UK doctors and a UK healthcare system where barriers to accessing healthcare and the financial and cultural implications of instituting the ASRS tool may not be equivalent in other healthcare systems.

It appears that ADHD in doctors is often under-diagnosed as shown by a high prevalence on incidental screening of a large cohort of UK doctors. The high rates of co-occurring anxiety and depression among people who screened positive for ADHD supports the importance of detailed assessments for ADHD in the group with mental health problems. Clinical pathways for triaging people at-risk and undertaking diagnostic assessments to understand mental health problems related to ADHD is a crucial aspect of supporting people with mental health problems. Adopting the ASRS is a simple, validated method of screening for ADHD and may have utility as a low-cost method of directing healthcare professionals towards appropriate psychiatric referral and evaluation. This study adds to the case for advocating for adequate mental health support and the importance of considering neurodevelopmental disorders in doctors. Future studies exploring ADHD in other health professional groups may help to understand neurodevelopmental disorder burden and its impact.
